# Shear Bond Strength of TheraCal PT and Calcium Silicate-Based Cement to a Flowable Composite Resin According to Bonding Strategy

**DOI:** 10.3390/jfb17090465

**Published:** 2026-09-10

**Authors:** Soram Oh

**Affiliations:** Department of Conservative Dentistry, College of Dentistry, Kyung Hee University, Seoul 02447, Republic of Korea; soram@khu.ac.kr

**Keywords:** calcium silicate-based cement, dental pulp capping, etch-and-rinse, self-etch, shear bond strength, universal dentin adhesive

## Abstract

This study aimed to evaluate the micro-shear bond strength (μSBS) of TheraCal PT (TP), Endocem MTA Premixed Heavy (EC), One-Fil Putty (OF), and BC Universal RRM (RM) bonded to a flowable composite resin using two universal adhesives (G-Premio Bond or Prime & Bond Universal) in two etching modes (etch-and-rinse or self-etch). Disc-shaped specimens (n = 60 per pulp capping material) were allowed to set for 72 h and then divided into four groups according to the adhesive and etching mode used. The μSBS was measured using a universal testing machine. Data were analyzed with three-way ANOVA and post hoc tests for the pulp capping material. Failure modes were determined. Spectra were recorded before and after adhesive application using attenuated total reflectance–Fourier transform infrared (ATR-FTIR) spectroscopy. Three-way ANOVA revealed that the effects of the adhesive (*p* = 0.002), etching mode (*p* < 0.001), and pulp capping material (*p* < 0.001) on the μSBS were significant. The interaction between the adhesive and etching mode, as well as the interaction between the adhesive and pulp capping material, was significant (*p* < 0.001). TP exhibited higher μSBS values than EC, OF, and RM. TP failed in mixed, cohesive, and adhesive modes, whereas EC, OF, and RM failed mostly cohesively within the material. ATR-FTIR spectroscopy confirmed the functional monomer peaks on the material surfaces after adhesive application.

## 1. Introduction

Vital pulp therapy (VPT) aims to preserve pulp vitality in both mature and immature teeth after pulp exposure resulting from caries removal, cavity preparation, or trauma. The 2-year success rate of partial or full pulpotomy in mature permanent teeth with deep caries is reported to be 85–98% when calcium silicate-based cement (CSBC) is used [1,2,3]. The causes of failure included residual infection, secondary caries, and microleakage [2,4]. Microleakage provides a passageway for pathogens, triggering clinical complications and acting as a risk factor for pulpal inflammation [5]. A tight bond between the pulp capping material and the restorative material, as well as between the pulp capping material and dentin, is an important factor in preventing microleakage [6].

Mineral trioxide aggregate (MTA) offers excellent sealing ability, biocompatibility, bioactivity, and moisture tolerance [7,8]. However, its drawbacks include a long setting time, poor handling characteristics, and potential for tooth discoloration [8,9,10]. Biodentine (Septodont, Saint-Maur-des-Fossés, France) contains zirconium oxide instead of bismuth oxide and uses calcium chloride as the setting accelerator. Therefore, it avoids discoloration, sets more rapidly, and is easier to handle than MTA [11]. Nevertheless, the use of Biodentine requires mixing equipment and procedures [12]. Recently, premixed CSBCs including Endocem MTA Premixed Heavy (EC; Maruchi, Wonju, Republic of Korea), One-Fil putty (OF; Mediclus, Cheongju, Republic of Korea), and BC Universal RRM (RM; Vericom, Chuncheon, Republic of Korea) have been introduced. These materials use zirconium (di)oxide as a radiopacifier to prevent discoloration and are supplied as single-component pastes that do not require mixing.

TheraCal PT (TP; Bisco, Schaumburg, IL, USA) is a dual-cured resin–modified calcium silicate cement that offers greater biocompatibility than its predecessor, TheraCal LC (Bisco, Schaumburg, IL, USA) [13]. Unlike the light-cure-only TheraCal LC, TP relies on a dual-curing mechanism that minimizes unreacted monomer release and thereby improves biocompatibility [13]. TP is supplied in a two-component auto-mix syringe and can be used for pulpotomy and pulp capping [14].

In VPT procedures, dentin adhesives are applied to the prepared tooth and the CSBC surface, followed by composite resin placement [15]. Universal adhesives can be used in either the etch-and-rinse (ER) or self-etch (SE) mode [12]. Kumar et al. reported higher shear bond strength (SBS) of MTA and Biodentine in ER than in SE mode [16]. Naiboğlu et al. also found higher SBS with ER mode for ProRoot MTA (Dentsply Tulsa Dental, Johnson City, TN, USA) and MTA Angelus (Angelus, Londrina, PR, Brazil) [12]. In contrast, Değer et al. and Baena et al. observed no significant difference in SBS of Biodentine between ER and SE modes [17,18]. To date, no studies have examined the SBS of premixed-type CSBCs or TP according to bonding strategy (ER vs. SE).

Fourier transform infrared (FTIR) spectroscopy is an analytical technique that measures the absorption of infrared radiation by molecules to identify their chemical composition and molecular structure [19]. When equipped with an attenuated total reflection (ATR) accessory, FTIR enables direct surface analysis without requiring powdered samples. FTIR spectroscopy has been used to monitor the setting reactions of hydraulic CSBC [20]. FTIR has also been used to measure the intensity of monomer–calcium salt formation after applying dental adhesives to tooth structures [21]. Calcium ions in CSBC may interact with the functional monomers of universal adhesives [22], yet the associated chemical structural changes have not been elucidated. Therefore, the present study used FTIR to investigate the structural changes at the surface of pulp capping materials following the application of a universal dentin adhesive.

The primary objective of this study was to evaluate the micro-shear bond strength (μSBS) of four pulp capping materials (EC, OF, RM, and TP) bonded to a flowable composite resin using different bonding strategies and to compare μSBS among the four materials. The secondary objective was to analyze the adhesive-coated surfaces of the pulp capping materials using FTIR and to investigate the interaction of the universal adhesive with hydrated CSBCs and polymerized TP.

The null hypotheses tested were that (1) the adhesive type, etching mode, and pulp capping material would not affect the μSBS to a flowable composite resin and (2) the FTIR spectra of the universal adhesive would not differ across the different pulp capping materials or etching modes.

## 2. Materials and Methods

### 2.1. Materials

The pulp capping materials tested were EC, OF, RM and TP. TP is polymerized via dual curing (light and chemical), whereas EC, OF and RM set via a hydration reaction. Scotchbond Universal Etchant (Solventum, St. Paul, MN, USA) was used in the ER groups. G-Premio Bond (GC Corp., Tokyo, Japan) and Prime & Bond Universal (Dentsply Sirona, Charlotte, NC, USA) were used as adhesives, and Unifil Lo Flo Plus A2 shade (GC Corp., Tokyo, Japan) was used as a flowable composite resin. The compositions of all materials are listed in Table 1.

### 2.2. Micro-Shear Bond Strength Test

The sample size was calculated using G*Power (version 3.1.9.4; Heinrich-Heine-Universität Düsseldorf, Düsseldorf, Germany). Based on a 2 × 2 × 4 factorial design with 16 groups, an a priori F test for analysis of variance (ANOVA: fixed effects, main effects, and interactions) was conducted with an effect size (f) of 0.25, α = 0.05, power (1 − β) = 0.9, and numerator df = 3. The required minimum total sample size was 231 specimens. Consequently, 15 specimens per group (240 specimens in total) were prepared to maintain a balanced design across all 16 groups.

**Figure 1:**
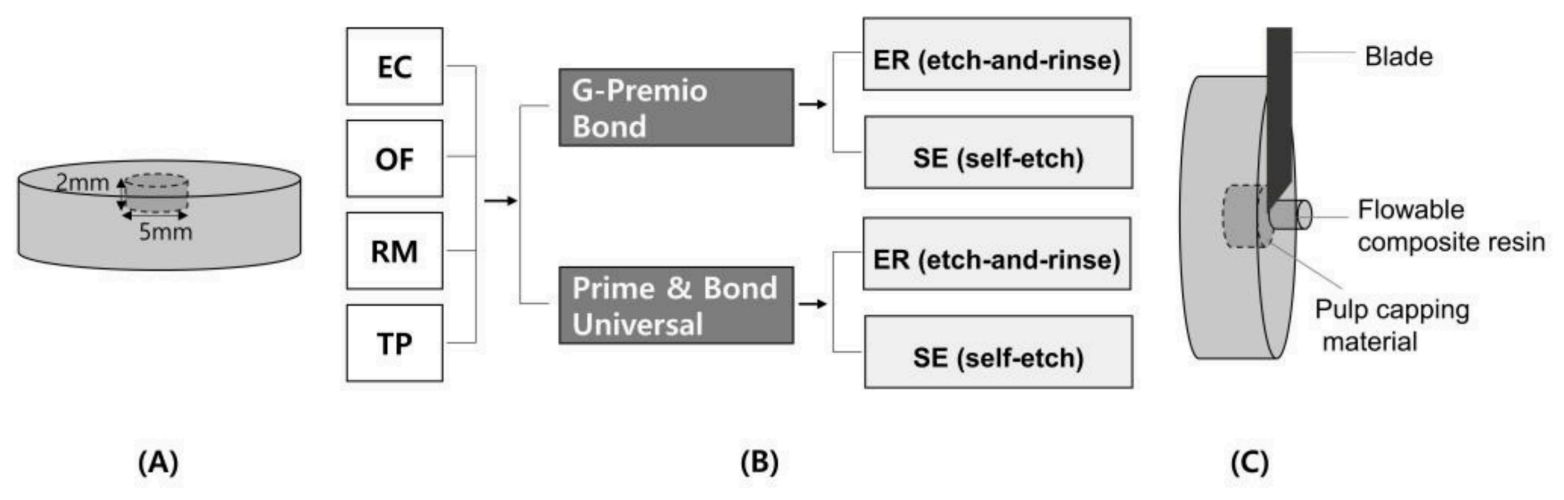
Experimental setup and groups of this study. (**A**) Disc-shaped specimen; (**B**) four tested pulp capping materials, and two universal adhesives, and two etching modes; (**C**) micro-shear bond strength (μSBS) test setup. EC, Endocem MTA Premixed Heavy; OF, One-Fil putty; RM, BC Universal RRM; TP, TheraCal PT.

Disc-shaped resin blocks (25 mm diameter, 5 mm thickness) with a central hole (5 mm, diameter, 2 mm depth) were three-dimensionally printed (n = 240; Figure 1A). The holes were filled with pulp capping material (n = 60 for each of EC, OF, RM and TP). TP was injected from its syringe tip and light-cured for 10 s with a light-emitting diode (LED) curing unit (Elipar DeepCure-S; Solventum, St. Paul, MN, USA), which has a 10 mm tip, a wavelength of 430–480 nm, an irradiance of 1470 mW/cm^2^, and an energy density of 14.7 J/cm^2^. OF was applied with a resin applicator. EC was dispensed directly from its proprietary syringe; RM was dispensed from its capsule. Based on the findings of a previous study on the same four pulp capping materials, the specimens were stored for 72 h at 95% relative humidity to ensure sufficient setting and maturation of the tested materials [23].

The specimens were divided into four subgroups according to their bonding strategy: G-Premio Bond (ER or SE) and Prime & Bond Universal (ER or SE) (Figure 1B). The assigned adhesive was applied to the pulp capping material according to the manufacturer’s instructions and light-cured for 10 s with the same LED curing unit (Elipar DeepCure-S, 14.7 J/cm^2^). A Tygon tube (internal diameter 1.59 mm, height 2 mm; ACF00002, Saint-Gobain, Courbevoie, France) was then positioned over the adhesive-coated material; a flowable composite resin (Unifil Lo Flo Plus A2) was placed inside the tube and light-cured for 20 s (29.4 J/cm^2^). After light curing, the specimens were stored for 24 h at 95% relative humidity.

The μSBS was measured using a universal testing machine (AGS-X; Shimadzu, Kyoto, Japan) at a crosshead speed of 0.5 mm/min. A knife-edge blade was applied onto each specimen until failure occurred at the interface between the flowable composite resin and CSBC or TP (Figure 1C). μSBS values were calculated in megapascals (MPa) using the following formula: μSBS (MPa) = load at failure (N)/area of the bonded surface (πr^2^) (mm^2^), where r is the internal radius of the Tygon tube in mm.

After the μSBS test, the debonded surfaces were examined under a stereomicroscope at ×20 magnification. The failure modes were classified as adhesive failure at the pulp capping material–flowable composite resin interface, cohesive failure within the flowable composite resin, cohesive failure within the pulp capping material, or mixed failure (partial cohesive and adhesive). Subsequently, representative specimens were platinum-coated and analyzed using field-emission scanning electron microscopy (FE-SEM; Apero S, Thermo Fisher Scientific, Waltham, MA, USA) at ×100 magnification.

### 2.3. Statistical Analysis

Data normality was assessed with the Shapiro–Wilk test and homogeneity of variances with Levene’s test. A three-way ANOVA was performed to evaluate the effects of adhesive, etching mode, and pulp capping material, as well as their interactions, on the μSBS, with Tukey’s test applied for post hoc comparisons among the pulp capping materials. All analyses were performed with IBM SPSS 25.0 (IBM Corp., Armonk, NY, USA) at a significance level of 95%.

### 2.4. FTIR Analysis

Infrared spectra were acquired with a FTIR spectrometer (Invenio-R; Bruker, Ettlingen, Germany) equipped with an ATR system. Each pulp capping material (EC, OF, RM, and TP) was dispensed onto a polyethylene film and pressed with a glass slide to obtain discs of 10 mm in diameter (n = 6 per material). The spectra were recorded 1 and 72 h after material placement. At 72 h, additional spectra were obtained after applying G-Premio Bond or Prime & Bond Universal, each used in either the ER or SE mode. Additionally, FTIR spectra were obtained for G-Premio Bond and Prime & Bond Universal after dispensing onto a Mylar strip, gentle air-blowing, and light-curing for 10 s with an LED curing unit (Elipar DeepCure-S; 14.7 J/cm^2^). All FTIR spectra were collected in triplicate over the range of 4000–400 cm^−1^ at 4 cm^−1^ resolution with 64 scans.

## 3. Results

### 3.1. Micro-Shear Bond Strength

Table 2 presents the mean μSBS values of the pulp capping materials bonded to the flowable composite resin.

Three-way ANOVA revealed that the effects of adhesive (*p* = 0.002), etching mode (*p* < 0.001), and pulp capping material (*p* < 0.001) on the μSBS were statistically significant (Table 3). The interaction between adhesive and etching mode, as well as the interaction between adhesive and pulp capping material, was statistically significant (*p* < 0.001). Neither the interaction between etching mode and pulp capping material (*p* = 0.079) nor that among adhesive, etching mode, and pulp capping material was statistically significant (*p* = 0.091). According to Tukey’s post hoc test results, TP exhibited the highest μSBS (*p* < 0.001), followed by EC and RM, which did not differ significantly; OF recorded the lowest μSBS (*p* < 0.001).

**Figure 2:**
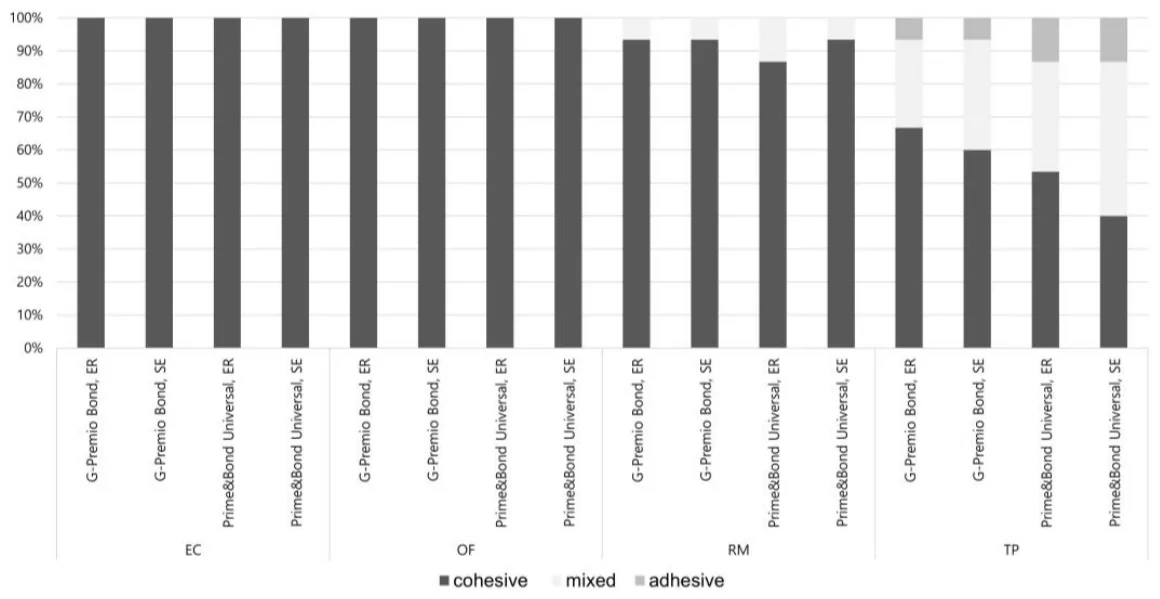
Distribution of failure patterns after the micro-shear bond strength (μSBS) test of the four pulp capping materials.

**Figure 3:**
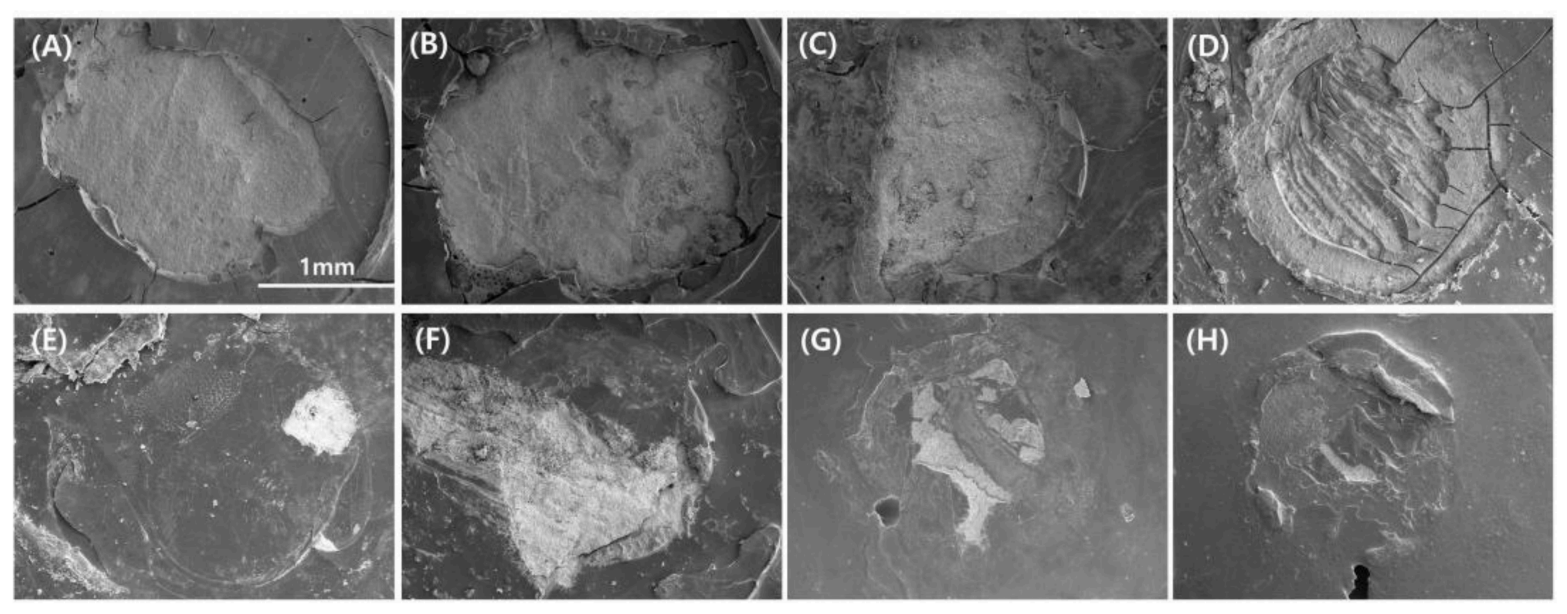
Representative field-emission scanning electron microscopy photographs of fractured surfaces. (**A**) Cohesive failure of EC, G-Premio Bond, SE; (**B**) cohesive failure of OF, G-Premio Bond, ER; (**C**) cohesive failure of RM, G-Premio Bond, SE; (**D**) cohesive failure of TP, Prime & Bond Universal, SE; (**E**) mixed failure of RM, Prime & Bond Universal, SE; (**F**) mixed failure of RM, Prime & Bond Universal, ER; (**G**) mixed failure of TP, G-Premio Bond, SE; (**H**) adhesive failure of TP, Prime & Bond Universal, SE.

Failure mode distributions are shown in Figure 2. EC and OF failed cohesively under all conditions. RM failed predominantly cohesively, with one or two specimens in each subgroup demonstrating mixed failure. In contrast, TP exhibited cohesive, adhesive, and mixed failures. TP specimens bonded with G-Premio Bond demonstrated 9–10 cohesive, 4–5 mixed, and 1 adhesive failure, while those bonded with Prime & Bond Universal presented 6–8 cohesive, 5–7 mixed, and 2 adhesive failures. Representative FE-SEM images of the failure patterns are presented in Figure 3.

### 3.2. FTIR Analysis

**Figure 4:**
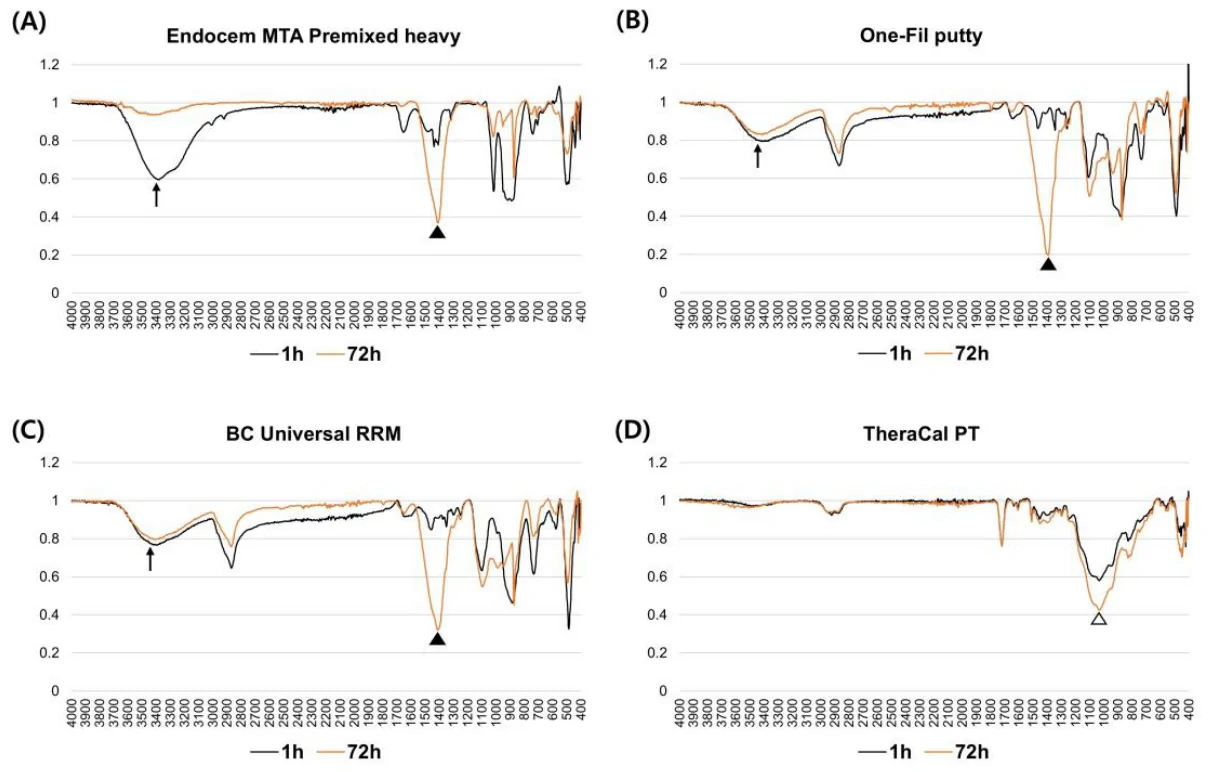
FTIR spectra of the four pulp capping materials 1 h and 72 h after application to the polyethylene film. (**A**) Endocem MTA Premixed Heavy (EC), (**B**) One-Fil Putty (OF), (**C**) BC Universal RRM (RM), (**D**) TheraCal PT (TP). An arrow indicates the O–H peak at 3100–3600 cm^−1^; a filled arrowhead indicates the carbonate peak at 1400–1450 cm^−1^; an unfilled arrowhead in the TheraCal PT panel indicates Si–O stretching at 900–1100 cm^−1^.

The FTIR spectra of the four pulp capping materials obtained 1 and 72 h after placement are shown in Figure 4. For EC, the broad O–H band at 3100–3600 cm^−1^ [20], evident at 1 h, flattened by 72 h, indicating the rapid consumption of free water during hydration (Figure 4A). After 72 h of hydration, EC, OF, and RM exhibited peaks in the 1400–1450 cm^−1^ region, reflecting carbonation [24], in which calcium hydroxide was converted into calcium carbonate (Figure 4A–C). For EC, OF, and RM samples, the Si–O/O–Si–O stretching band at 800–1100 cm^−1^ [25,26,27] weakened from 1 h to 72 h, indicating the hydration of calcium silicates into an amorphous calcium silicate hydrate gel matrix (Figure 4A–C). In TP, the O–H peak at 3100–3600 cm^−1^ was absent, and the sharp C=O carbonyl peak at 1720 cm^−1^ [28] persisted for 72 h after placement, reflecting the resin-modified silicate matrix (Figure 4D). The absorption band at 900–1100 cm^−1^ deepened by 72 h, indicating the post-curing maturation of the Si–O network.

**Figure 5:**
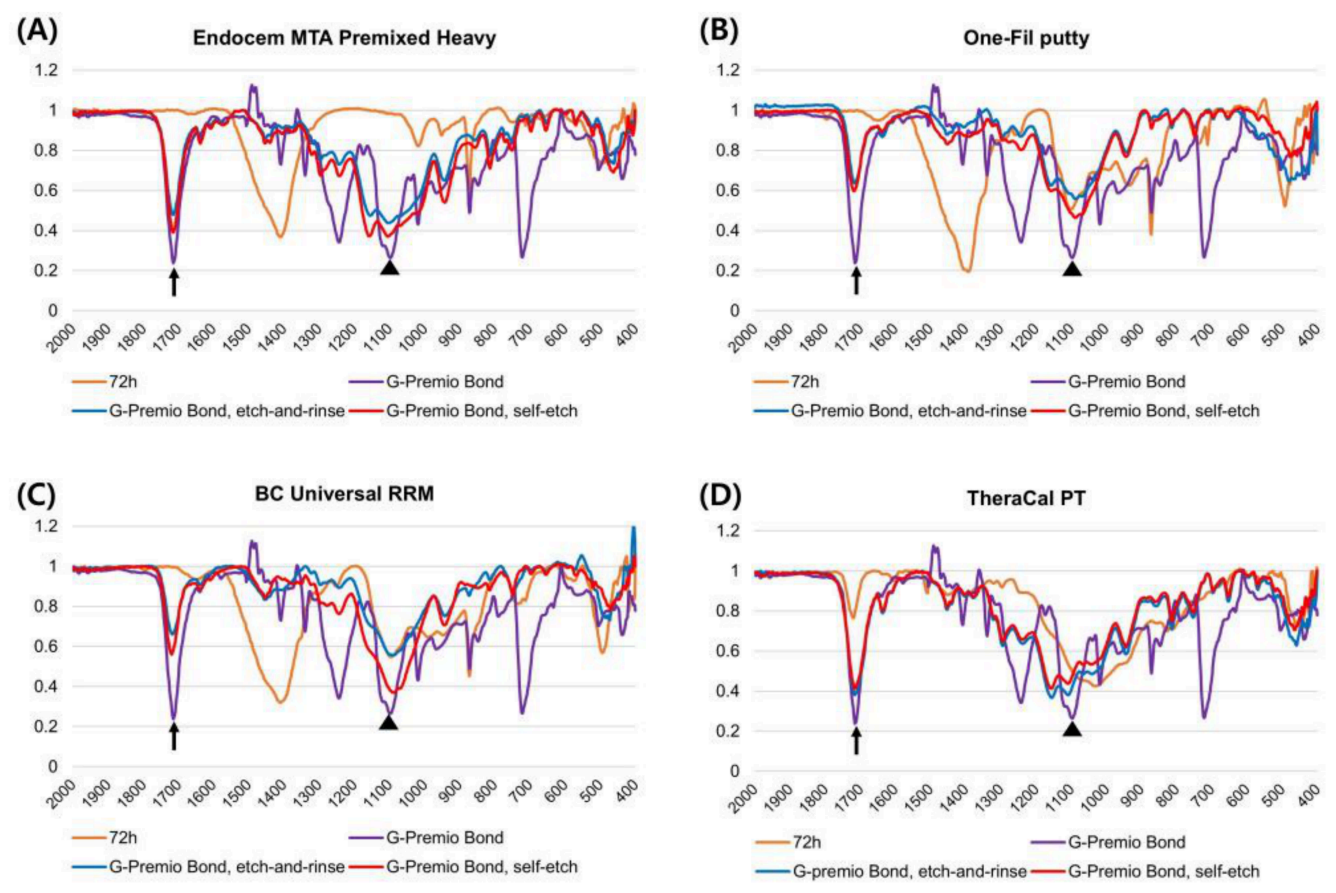
Overlaid FTIR spectra of the four pulp capping materials obtained 72 h after post-placement, after applying G-Premio Bond in ER or SE mode, and of G-Premio Bond alone. (**A**) Endocem MTA Premixed Heavy (EC), (**B**) One-Fil Putty (OF), (**C**) BC Universal RRM (RM), (**D**) TheraCal PT (TP). An arrow indicates carbonyl (C=O) peaks at 1718 cm^−1^; a filled arrowhead indicates the functional phosphate monomer region at 1100 cm^−1^.

Overlaid FTIR spectra of the four pulp capping materials after 72 h, after application of G-Premio Bond in ER or SE mode, and of G-Premio Bond alone over the wavenumber range of 2000–400 cm^−1^ are shown in Figure 5. The prominent carbonyl stretching peak (C=O) at 1718 cm^−1^ (arrows), attributable to 10-methacryloyloxydecyl dihydrogen phosphate (10-MDP) [29,30], of G-Premio Bond appeared in all materials after application to the pulp capping materials (Figure 5A–D), although its intensity was visibly lower for OF and RM. The peak at 1100 cm^−1^ (arrowhead), assigned to C–O bond stretching or phosphate group vibration [29,31,32], indicative of the functional phosphate monomer region of G-Premio Bond, split into two peaks (1100 and 1155 cm^−1^) for EC and TP (Figure 5A,D), suggesting the formation of chemical bonds.

**Figure 6:**
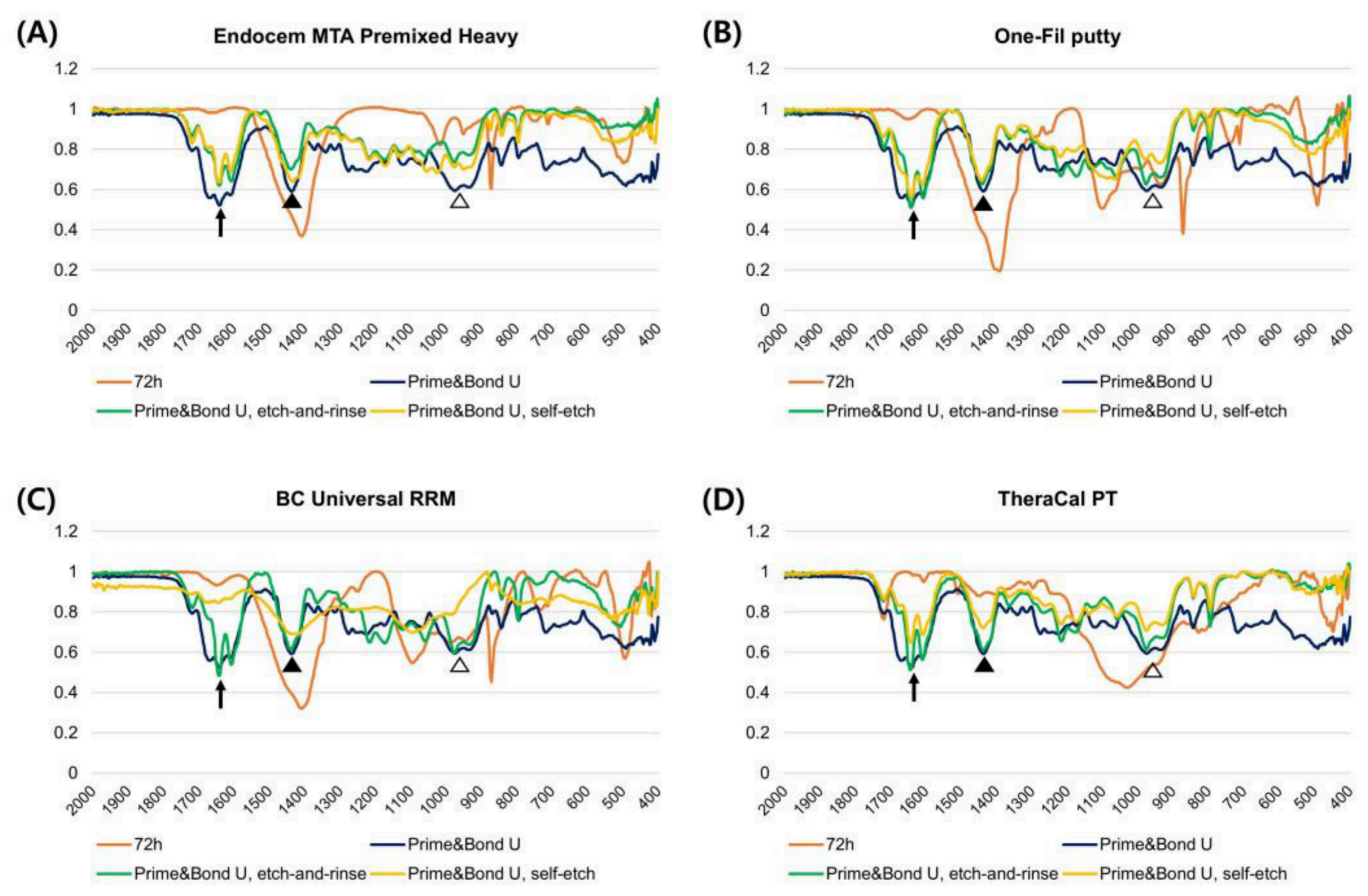
Overlaid FTIR spectra of the four pulp capping materials obtained 72 h after post-placement, after applying Prime & Bond Universal in ER or SE mode, and of Prime & Bond Universal alone. (**A**) Endocem MTA Premixed Heavy (EC), (**B**) One-Fil Putty (OF), (**C**) BC Universal RRM (RM), (**D**) TheraCal PT (TP). An arrow indicates the aliphatic C=C stretching peak at 1640 cm^−1^; a filled arrowhead indicates the C–H bending peak at 1436 cm^−1^; an unfilled arrowhead indicates the 10-MDP region at 954–991 cm^−1^.

Overlaid FTIR spectra of the four pulp capping materials after 72 h, after application of Prime & Bond Universal in ER or SE mode, and of Prime & Bond Universal alone over the wavenumber range of 2000–400 cm^−1^ are shown in Figure 6. The peak at 1640 cm^−1^ (aliphatic C=C stretching) of Prime & Bond Universal split into two peaks on all pulp capping materials in the ER mode (green line), indicating the formation of chemical bonds, and the intensity was stronger for OF, RM, and TP than for EC. Prime & Bond Universal demonstrated a peak at 1436 cm^−1^, assigned to C–H bending of the methacrylate monomer [29,33], which was maintained for all pulp capping materials after application of the adhesive without a shift (Figure 6A–D). When Prime & Bond Universal was applied in the ER mode, the increased intensity of the spectrum at 954–991 cm^−1^ closely matched that reported for 10-MDP by Almusa et al. [34] across OF, RM, and TP substrates, but was attenuated in the SE mode (yellow line) (Figure 6B–D). However, for EC, the peak intensity at 954–991 cm^−1^ in the ER mode was weaker than that in the SE mode (Figure 6A).

## 4. Discussion

The adhesive type, etching mode, and pulp capping material all had a significant effect on the μSBS between the pulp capping materials and the flowable composite resin. Therefore, the first null hypothesis, that the adhesive type, etching mode, and pulp capping material would not affect the μSBS to a flowable composite resin, was rejected. Furthermore, significant interactions were observed between adhesive and etching mode and, between adhesive and pulp capping material, indicating that the μSBS was dependent on both the specific pulp capping material used and the bonding strategy. These findings indicate that achieving optimal bond strength between the pulp capping material and the flowable composite resin requires selecting an appropriate adhesive, with the choice of etching mode depending on the selected adhesive.

The interaction between adhesive and etching mode on the μSBS was statistically significant. Prime & Bond Universal is a mildly acidic universal adhesive (pH 2.5–2.7) that contains 10-MDP and dipentaerythritol penta-acrylate phosphate (PENTA) as acidic functional monomers [35]. 10-MDP superficially dissolves hydroxyapatite and deposits water-insoluble 10-MDP–Ca salts, thereby establishing stable ionic bonding [36]. PENTA is a hydrophilic monomer that promotes decalcification rather than chemical adhesion [37]. Prime & Bond Universal seems to be more suitable for the ER mode than the SE mode because phosphoric acid etching compensates for the weak chemical bonding of PENTA [37]. In contrast, G-Premio Bond is an intermediately strong adhesive with a pH of 1.5. The greater acidity of G-Premio Bond may produce more pronounced demineralization, which may minimize the difference in μSBS between the ER and SE modes [38].

Consistent with previous studies, TP exhibited the highest μSBS among the four materials tested [39,40]. As a dual-cured resin-modified calcium silicate material, TP may form strong chemical interactions with bonding agents. FTIR spectra indicated a pronounced carbonyl (C=O) peak at 1720 cm^−1^ across all four adhesive-mode combinations. Among the premixed-type CSBCs, EC and RM demonstrated higher μSBS values than OF. These results may stem from the compositional differences among premixed-type CSBCs, such as water content, thickening agents, and zirconium oxide proportions. The FTIR spectra revealed a substantial decrease in the O–H band (3100–3600 cm^−1^) in EC after 72 h, whereas OF and RM exhibited minimal changes in this region. Conversely, the carbonate peak (1400–1450 cm^−1^) increased most prominently in OF after 72 h. Whether this indicates greater reaction-product formation in OF requires further investigation.

EC and OF failed entirely cohesively; RM failed predominantly cohesively within the pulp capping materials, whereas TP showed mixed and adhesive failure patterns as well as cohesive failures. In general, the groups with lower bond strengths presented higher proportions of cohesive failure within the pulp capping material. μSBS testing may reflect the intrinsic mechanical resistance of the substrate rather than pure interfacial bond failure, leading to fracture within the weaker material [41,42,43]. Therefore, the μSBS values should be interpreted with caution [44].

In this study, all pulp capping materials were allowed to mature for 72 h before the adhesive procedure. A previous study [23] evaluated the push-out bond strength and performed scanning electron microscopic analysis of the same four pulp capping materials (TP, EC, OF and RM) and revealed that applying a universal adhesive (Prime & Bond Universal in SE mode) 1 h after placement resulted in less crystalline formation within the subsurface dentinal tubules compared with 72 h post-placement across all four materials. Additionally, phase separation occurred when the universal adhesive was applied 1 h after the placement of EC, OF, and RM. In contrast, the universal adhesive was evenly distributed on the CSBC surface 3 days after placement. Consequently, a 72 h storage period was selected in the present study to ensure complete setting and maturation of the tested materials.

This study has several limitations. First, the bonding performance was evaluated exclusively through short-term μSBS testing without long-term aging protocols. Although immediate testing provides essential baseline data on initial interfacial integrity, it does not account for the hydrolytic degradation of the adhesive or the long-term hydration dynamics of CSBCs [45]. Second, because nominal μSBS values are influenced by stress concentration and the materials’ elastic moduli [46], they represent overall resistance to shear forces rather than absolute interfacial molecular adhesion. Future studies involving artificial aging are required to evaluate long-term clinical durability [47].

The FTIR peak at 954–991 cm^−1^ obtained by application of Prime & Bond Universal (Figure 6) closely matched the reported spectrum of 10-MDP [34]. And the present study revealed an enhanced FTIR peak at carbonyl stretching (1718 cm^−1^) and in the phosphate monomer region (1100–1155 cm^−1^) after application of G-Premio Bond to the pulp capping materials (Figure 5). A prior study [30] reported a carbonyl stretching peak at 1718 cm^−1^ observed during 10-MDP chemisorption to enamel. Similar reactions of 10-MDP with CSBC and enamel support the chemical coupling between the CSBC-derived calcium and the universal adhesive.

A limitation of FTIR analysis is that it cannot confirm the chemical bonding between the calcium ions and universal adhesives [48]. The spectral peaks correspond to functional groups that may originate from multiple sources. Therefore, the peak assignments in the FTIR analysis must be interpreted cautiously. Transmission electron microscopy, nuclear magnetic resonance, and X-ray diffraction are required to confirm the interactions between the functional monomers and calcium ions [48]. Because ATR-FTIR spectra were collected from the material surface, the degree of chemical cure and internal hydration in the FTIR samples may differ from that in the μSBS test specimens.

Moreover, in the present study, the adhesive was applied 72 h after the placement of the pulp capping materials; therefore, these results cannot be directly extrapolated to clinical situations in which the flowable composite resin restoration is bonded during the same visit as the VPT procedure. When selecting pulp capping material, factors such as biocompatibility, sealing ability, and the capacity to induce tertiary dentin should be considered.

## 5. Conclusions

The micro-shear bond strength between the pulp capping material and the flowable composite resin was significantly affected by the adhesive, etching mode, and type of pulp capping material. Statistically significant interactions were observed between the adhesive and the etching mode and between the adhesive and the pulp capping material. Consequently, in vital pulp therapy, the choice of pulp capping material can influence the compatible adhesive system, while the selected adhesive determines the appropriate etching mode. The FTIR spectra after adhesive application differed depending on the bonding strategy and pulp capping material. The FTIR spectra supported the chemical reaction between the functional monomers of the universal adhesive and the calcium ion of CSBCs.

## Figures and Tables

**Table 1:** Materials and compositions used in this study.

Product (Code), Lot Number	Manufacturer	Composition	Steps for Application
**Endocem MTA Premixed Heavy (EC), QD260326**	Maruchi, Wonju, Republic of Korea	Zirconium dioxide, calcium silicate, calcium aluminate, calcium sulfate, dimethyl sulfoxide, thickening agent	
**One-Fil putty (OF), OP63T373**	Maruchi, Wonju, Republic of Korea	Zirconium dioxide, calcium silicate, calcium aluminate, calcium sulfate, dimethyl sulfoxide, thickening agent	
**BC Universal RRM** **(RM), WT453200**	Vericom, Chuncheon, Republic of Korea	Calcium aluminosilicate compound, zirconium oxide, thickening agent	
**TheraCal PT** **(TP), 2600001548**	Bisco, Schaumburg, IL, USA	Base: Silicate glass-mixed cement, Bis-GMA, Barium Zirconate Catalyst: Bis-GMA, Triethylene Glycol Dimethacrylate, Barium Zirconate, Ytterbium Fluoride, Benzoyl Peroxide	Light cure for 10 s
**Scotchbond Universal Etchant Etching Gel, 12986332**	Solventum, St. Paul, MN, USA	32% phosphoric acid	Apply the etching gel to the precise area and allow to react for 15 s, rinse thoroughly for 15 s with water.
**G-Premio Bond, 2601131**	GC Corp., Tokyo, Japan	MDP, 4-MET, 2-hydroxy-1,3-dimethacryloxypropanacetone, 2,6-di-tert-butyl-p-cresol, acetone, water, photoinitiator, silica filler	Apply bond and wait for 10 s, dry for 5 s, light-cure for 10 s.
**Prime & Bond Universal, 2510000419**	Dentsply Sirona, Charlotte, NC, USA	PENTA, 10-MDP, Active Guard Technology crosslinker, photoinitiator, stabilizer, isopropanol, water	Apply bond and agitate for 20 s, evaporate solvent for 5 s, light-cure for 10 s.
**Unifil Lo Flo Plus A2 shade, 2512011**	GC Corp., Tokyo, Japan	Fluoro-aluminosilicate glass, organic filler colloidal silica, urethane dimethacrylate, dimethacrylate, photo-initiators, stabilizer/63 wt%	Light-cure for 10 s with LED at 1200 mW/cm^2^

Bis-GMA, bisphenol-A-glycidyldimethacrylate; MDP, methacryloyloxydecyl dihydrogen phosphate; 4-MET, 4-methacryloyloxyethyl trimellitate; PENTA, dipentaerythritol pentacrylate phosphate; LED, light-emitting diode.

**Table 2:** Micro-shear bond strength (MPa) of the four pulp capping materials according to bonding strategy (mean ± standard deviation).

	EC	OF	RM	TP
**G-Premio Bond, ER**	8.0 ± 1.6	3.8 ± 1.4	6.6 ± 1.4	10.3 ± 2.4
**G-Premio Bond, SE**	6.8 ± 1.4	4.0 ± 1.0	5.0 ± 1.0	11.6 ± 2.6
**Prime & Bond Universal, ER**	7.7 ± 1.6	5.3 ± 1.6	7.2 ± 1.6	15.5 ± 2.1
**Prime & Bond Universal, SE**	4.7 ± 1.4	3.1 ± 1.0	5.3 ± 1.0	13.1 ± 2.7

EC, Endocem MTA Premixed Heavy; ER, etch-and-rinse; OF, One-Fil Putty; RM, BC Universal RRM; SE, self-etch; TP, TheraCal PT.

**Table 3:** Results of the three-way analysis of variance (ANOVA) for micro-shear bond strength.

Source	DF	Mean Square	F	*p* Value	$\eta_{p}^{2}$
**Adhesive**	1	32.634	9.693	0.002	0.041
**Etching mode**	1	107.870	32.040	<0.001	0.125
**Pulp capping material**	3	811.778	241.120	<0.001	0.764
**Adhesive × etching mode**	1	64.377	19.122	<0.001	0.079
**Adhesive × pulp capping material**	3	55.139	16.378	<0.001	0.180
**Etching mode × pulp capping material**	3	7.709	2.290	0.079	0.030
**Adhesive × etching mode × pulp capping material**	3	7.357	2.185	0.091	0.028

DF, degrees of freedom; $\eta_{p}^{2}$, partial eta squared.

## Data Availability

The raw data supporting the conclusions of this article will be made available by the author on reasonable request.

## Abbreviations

The following abbreviations are used in this manuscript:

ATR	Attenuated total reflectance
CSBC	Calcium silicate-based cement
EC	Endocem MTA Premixed Heavy
ER	Etch-and-rinse
FE-SEM	Field-emission scanning electron microscopy
FTIR	Fourier transform infrared
LED	Light-emitting diode
OF	One-Fil Putty
RM	BC Universal RRM
SBS	Shear bond strength
SE	Self-etch
TP	TheraCal PT
VPT	Vital pulp therapy
